# Expanding the Molecular Genetic Landscape of Dystrophinopathies and Associated Phenotypes

**DOI:** 10.3390/biomedicines12122738

**Published:** 2024-11-29

**Authors:** Katja Neuhoff, Ozge Aksel Kilicarslan, Corinna Preuße, Ann-Kathrin Zaum, Heike Kölbel, Hanns Lochmüller, Ulrike Schara-Schmidt, Kiran Polavarapu, Andreas Roos, Andrea Gangfuß

**Affiliations:** 1 Department of Pediatric Neurology, Centre for Neuromuscular Disorders, Centre for Translational Neuro- and Behavioral Sciences, University Duisburg-Essen, 45122 Essen, Germany; katja.neuhoff@uk-essen.de (K.N.); heike.koelbel@uk-essen.de (H.K.); ulrike.schara-schmidt@uk-essen.de (U.S.-S.); andreas.roos@uk-essen.de (A.R.); 2 Children’s Hospital of Eastern Ontario Research Institute, Ottawa, ON K1H 5B2, Canada; oakselkilicarslan@cheo.on.ca (O.A.K.); hlochmuller@toh.ca (H.L.);; 3 Department of Neuropathology, Charité-University Medicine Berlin, 10117 Berlin, Germany; corinna.preusse@charite.de; 4 Institute of Human Genetics, University of Würzburg, 97074 Würzburg, Germany; ann-kathrin.zaum@uni-wuerzburg.de; 5 Centro Nacional de Análisis Genómico (CNAG-CRG), Center for Genomic Regulation, Barcelona Institute of Science and Technology (BIST), 08028 Barcelona, Spain; 6 Brain and Mind Research Institute, University of Ottawa, Ottawa, ON K1N 6N5, Canada; 7 Division of Neurology, Department of Medicine, The Ottawa Hospital, Ottawa, ON K1H 8L6, Canada; 8 Department of Neuropediatrics and Muscle Disorders, Faculty of Medicine, Medical Center, University of Freiburg, 79106 Freiburg, Germany; 9 Department of Neurology, Medical Faculty, Heinrich Heine University Duesseldorf, 40225 Duesseldorf, Germany

**Keywords:** Becker muscular dystrophy, BMD, DMD, Duchenne muscular dystrophy, dystrophin, molecular diagnosis, novel pathogenic variants

## Abstract

**Background/Objectives**: X-linked dystrophinopathies are a group of neuromuscular diseases caused by pathogenic variants in the *DMD* gene (MIM *300377). Duchenne muscular dystrophy (DMD; MIM #310200) is the most common inherited muscular dystrophy. **Methods**: We screened datasets of 403 male, genetically confirmed X-linked dystrophinopathy patients and identified 13 pathogenic variants of the *DMD* gene that have not been described in the literature thus far. For all patients we provide additional data on the clinical course, genotype–phenotype correlations as well as histological datasets of nine patients. In two cases, we used RNA-Seq analyses, showing that this method can be particularly helpful in cases of deep intrinsic variants. **Results**: We were able to show, that a combination of the different datasets is helpful to counsel families and provides a better understanding of the underlying pathophysiology. **Conclusions**: Overall, we elaborated upon the persistent challenge of determining the course of disease from genetic analysis alone, rather supporting the concept of a clinical continuum of dystrophinopathies with our combined clinical, histological and molecular genetic findings.

## 1. Introduction

Duchenne muscular dystrophy (DMD) is the most common inherited muscular dystrophy in childhood, affecting one in 3500 to 5000 of male newborns [1,2]. Becker muscular dystrophy (BMD), representing its rarer form, which has a milder and slower disease course, affects one in about 18,000 male newborns [3,4]. The term intermediate muscular dystrophy (IMD) refers to patients whose course of disease is less severe than DMD but worse than BMD. Nowadays, there is an increasing assumption of a “clinical continuum” of both forms [5], and even female manifesting carriers have been described [6]. Taking these clinical aspects into consideration, one might designate individuals suffering from muscular diseases based on pathogenic variants affecting the *DMD* gene as dystrophinopathy patients.

Boys affected by dystrophinopathy clinically categorized as DMD show a weakness of the proximal muscles, especially in the shoulder and pelvic girdle, inter alia leading to a positive Gowers maneuver [7]. Typical early disease features are delayed motor milestones as well as elevated levels of serum creatine kinase (CK). Later, affected boys present with a broad-based gait and difficulty climbing stairs [8]. At an average age of 11–13 years, DMD patients lose the ability to walk independently and become wheelchair-dependent [8,9], while IMD patients remain ambulatory until up to 12–15 years [5]. In contrast, BMD patients lose their ability to walk at an average age of 38 years [10]. The combination of cardiac and respiratory involvement is commonly the cause of death at an average age of 32 years for DMD [11] and 30 to 40 years for IMD [12]; BMD patients frequently reach a normal life span [13].

The *DMD* gene is located at Xp21 [14], being the largest human gene (2.4 Mb) and consisting of 79 exons [15]. In 1986, the first pathogenic variants affecting this gene were described as the cause of Becker and Duchenne muscular dystrophies, respectively [16]. Deletions account for 68% of the pathogenic variants, 11% are duplications and 20% of patients have smaller pathogenic variants such as point mutations. Of these smaller variants, half are nonsense mutations [17]. Notably, X chromosomal inversion disrupting the *DMD* gene was also identified as the underlying genetic cause in some dystrophinopathy patients [18]. To date, more than 7000 causative variants have been described in the literature [17,19,20].

The product of the *DMD* gene, the dystrophin protein, consists of four domains which fulfill different functions: the N′ terminus binds to actin, which is followed by a rod-like region consisting of 24 spectrin-like repeats, which are interrupted at different intervals by four proline-containing sections [21], providing the flexibility of the protein [22]. Adjacent to the C’ terminus, a cysteine-rich region is localized [21]. Overall, as part of the dystrophin–glycoprotein complex, the dystrophin protein stabilizes the plasma membrane of the cell and facilitates the physical interaction of this membrane with the cytoskeleton [23]. Moreover, the dystrophin–glycoprotein complex plays an important role in the assembly and regulation of the activity of sarcolemmal ion channels, e.g., the potassium channel Kir4.1 [24]. Of note, the presence of seven different promoters and tissue-specific splicing results in the expression of different isoforms of dystrophin of varying lengths across different cellular populations [25].

For prognosis of the clinical course, Monaco’s frame shift hypothesis can be applied in about 92% of cases [26,27]. According to this hypothesis, an intact reading frame of the *DMD* gene leads to the milder form of BMD. However, if the reading frame of protein biosynthesis is shifted by the pathogenic variant, this leads to the clinical picture of DMD [27]. Exceptions are patients showing a milder clinical picture despite a shifted reading frame in addition to patients developing the clinical presentation of DMD despite an intact reading frame; this might be explained by the different functions of the individual domains of the protein. For example, deletion of the amino terminus leads to DMD, whereas deletion of part of the spectrin-like domain leads to BMD, even if neither deletion shifts the reading frame [28]. The severity of the phenotype also depends on the amount of functional dystrophin in muscle and other cellular populations such as neurons: the more functional dystrophin is expressed, the less severe is the phenotypical manifestation, with <3% dystrophin leading to DMD and >10% leading to BMD [29].

To establish the molecular genetic diagnosis, multiplex ligation-dependent probe amplification (MLPA) should be performed, detecting large deletions and duplications [30]. If MLPA is inconclusive, complete sequencing of the gene by next-generation sequencing should be performed to identify point mutations [31,32]. If the sequencing is also inconclusive, a muscle biopsy may allow for the drawing of further conclusions by studying dystrophin levels [33]. Along this line, the investigation of muscular dystrophin levels may also be helpful in the attempt to find genotype–phenotype correlations.

In this study, we report 13 novel substitutions and small insertion/deletions (INDELs) in the *DMD* gene not yet described in the literature and the associated phenotypes including the associated myopathology.

## 2. Materials and Methods

### 2.1. Editorial Policies and Ethical Considerations

This work was approved by the ethical committee of the University Duisburg-Essen (22-10521-BO). Written informed consent was obtained from each patient or the parents/caregivers (for patients < 18 years). This study was conducted in accordance with the principles of the Declaration of Helsinki.

### 2.2. Study Participants

We screened datasets of 403 male, genetically confirmed X-linked dystrophinopathy patients followed up at the Centre for Neuromuscular Diseases, part of the Department of Pediatric Neurology of the University Children’s Hospital Essen from 2008 to 2024. Clinical records were retrospectively reviewed for the type of mutation, demographic data, such as age and gender, as well as for clinical features and other disease-related elements: age of first symptom, type of first symptom, age at diagnosis, ventilatory support, motor milestone development, ambulatory status, heart and skeletal manifestation, and other comorbidities and interventions, as well as genetic testing and muscle biopsy findings (see Table 1 and Table 2).

Among these 403 patients, we identified 13 harboring putative disease-causing *DMD* substitutions and INDELs thus far not published in the literature or having insufficient evidence in current variant databases (Leiden Open Variation Database, LOVD, www.dmd.nl; Universal Mutation Database TREAT-NMD DMD, UMD-DMD, http://umd.be/TREAT_DMD/ and ClinVar database, ClinVar, https://www.ncbi.nlm.nih.gov/clinvar/, last accessed on 11 June 2024).

#### 2.2.1. Phenotype Predictions

We correlated the genetic findings with the observed phenotype of dystrophinopathy in these 13 patients. Doing so, we clinically predicted severe DMD vs. milder IMD/BMD phenotypes based on clinically verifiable outcomes (e.g., loss of ambulation and CK level) as listed in Supplementary Table S1.

#### 2.2.2. Molecular Genetic Findings

The molecular genetic findings of 13 patients were retrospectively obtained from DNA and/or RNA sequencing results. *DMD* gene variants were annotated using the longest canonical muscle transcript (NM_004006; Dp427m). In silico predictions of abnormal splicing were analyzed using SpliceAI [34] and Human Splicing Finder (HSF) (https://hsf.genomnis.com/, accessed on 1 August 2024; [35]). While loss and gain of putative splice sites were analyzed based on SpliceAI predictive scores and HSF predictions, abnormalities of splice regulatory elements (SREs) resulting in an imbalance of exon splicing enhancers (ESEs) and silencers (ESSs) were predicted using HSF. Variants were classified based on American College of Medical Genetics and Genomics (ACMG) criteria [36]. Further genotype–phenotype correlation was performed based on available clinicopathological data and variant predictions (for further details see Supplementary Document S2).

Given that this was a retrospective data analysis, the DNA testing for each patient was mostly carried out as step-by-step analytic procedure in the context of a routine diagnostic work-up. This meant that most patients of our entire dystrophinopathy cohort first underwent a multiplex ligation-dependent probe amplification (MLPA) analysis for deletions/duplications in the *DMD* gene. If this remained unremarkable, next, muscle biopsies were collected for microscopic investigation (before around 2015). If microscopic evaluation revealed a dystrophic pattern, the next step was a point mutation analysis of the *DMD* gene, in most patients, as targeted sequencing (before 2015). From around 2020, exome sequencing became increasingly established as an analytic method.

Monaco frame shift hypothesis criteria were applied to assess the molecular genetic findings.

## 3. Results

### 3.1. Overall Molecular Genetic Findings

**Figure 1:**
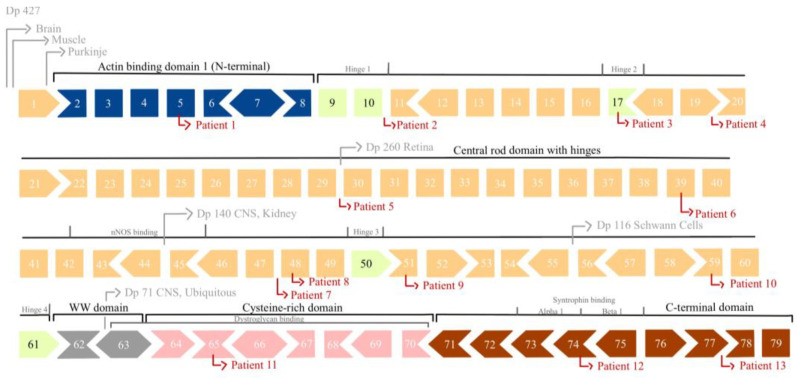
Schematic representation of the 79 exons of dystrophin and location of the novel pathogenic variants. Dark blue: actin-binding domain 1 (N-terminal domain), exons 2-8; yellow: central rod domain, exons 9-61 (including hinges (highlighted in lighter yellow, exons 9-10, 17, 50 and 61) and nNOS binding site, exons 42-45); gray: WW domain, exons 62-63; pink: cysteine-rich domain, exons 64-70; orange: C-terminal domain, exons 71-79 (including Syntrophin binding sites Alpha 1 and Beta 1, exons 73-75). The first exons of the different isoforms are marked by the labeled arrows, respectively. Rectangles indicate in-frame exons, whereas arrows indicate codons disrupted by exon junctions.

Data-based screening of genetic variants was carried out in the overall cohort of 403 male dystrophinopathy patients. Here, we identified 13 *DMD* small variants (substitutions and INDELs out of which six were exonic variants resulting in truncations (five frameshift and one nonsense)). A canonical splice site and missense variants were present in two patients each and two patients had deep intronic substitutions (see Figure 1 and Table 1). In all 13 cases, clinical manifestation accorded with the known phenotypical spectrum of a dystrophinopathy, in turn indicating their pathogenicity. Overall, these variants affected exons 5, 17, 39, 48, 51, 59, 65 and 74 as well as introns 10, 19, 29, 47 and 77, and were thus distributed across the entire *DMD* gene. Table 1 provides an overview of the molecular genetics and clinical findings of all 13 patients. The exonic variants causing frameshift or nonsense codon and canonical splice site variants were presumed to cause partial or total loss of functional DMD protein. However, deep intronic and missense variants were further analyzed to predict possible splicing defects.

#### 3.1.1. Deep Intronic Variants

In patients 2 and 13, RNA analysis identified deep intronic variants predicted to cause mis-splicing. Sequence analysis of cDNA revealed that patient 2 has a deep intronic variant (c.1149+273T>G,) in their *DMD* gene, which led to the activation of a cryptic splice site in intron 10, resulting in the creation of a 166 bp pseudoexon (Supplementary Figure S2). This in turn is predicted to have resulted in a stop codon and premature truncation within the pseudoexon (p.Gly384Leufs*3).

In patient 13, RNA-Seq from muscle tissue identified a rare novel deep intronic variant (c.11015-545A>G), which created a new splice acceptor site in intron 77 of the *DMD* gene, resulting in the inclusion of distal intron 77 into exon 78. The included intron had a premature stop codon, causing premature truncation within the new exon 78 (p.Gly3672Aspfs*73). The in silico predictions (SpliceAI and HSF) for both deep intronic variants correlated well with the observed pseudoexon formation (Supplementary Figure S2). These findings are compatible with clinical symptoms and the muscle biopsy in both patients.

#### 3.1.2. Missense Variants

In patients 1 and 11, rare missense variants were identified affecting exons 5 and 65, respectively. In patient 1, two-base pair INDEL c.336_337delinsTT resulted in a consecutive missense change p.Trp112_Asn113delinsCysTyr, while in patient 11, simple substitution c.9527A>G with corresponding amino acid substitution (p.Asp3176Gly) was observed, respectively. The c.9527A>G in patient 11 was predicted to cause a cryptic donor site by both SpliceAI and HSF along with an imbalance of SREs (HSF). The other two missense changes were not predicted as splice altering by SpliceAI but were predicted to result in an imbalance of SREs (ESE/ESS ratio) by HSF, which could have affected the normal splicing of the corresponding exons. Hence, all two missense variants identified in this study were predicted to have an effect on normal splicing.

### 3.2. Clinical Presentations

The analysis of the clinical data showed that four cases could be phenotypically categorized as BMD (1, 2, 12 and 13) and four (4, 8, 9 and 11) cases as DMD. This cannot yet be finally assessed for patients 3, 5, 6, 7 and 10 due to their actual ages.

Overall, the clinical results showed initial symptoms between 6 months and 10 years. Motor delay was the first symptom in 6/13 patients, 2/13 patients suffered from muscle pain, whereas exercise intolerance, abnormal gait, frequent falls and fine motor delay occurred in 1/13 patients each and the initial symptoms of one patient were unknown. Muscle biopsies were taken at an average age of 1 to 5 years, with CK results ranging from a minimum of 2.800 U/I to a maximum of 38.493 U/I. Treatment with Deflazacort was carried out in 7/13 patients, started at an average age of 5 to 7 years, five patients did not take any medication and one patient stopped medication with Deflazacort after one year. One patient was taking Translarna additionally. Ventilatory support was needed in none of the cases, cardiac involvement was observed in 4/13 cases and CNS involvement in 7/13 cases, including speech delay, motor tics, difficulties concentrating, autistic behavior as well as a global developmental delay. The present age of the patients ranged from 1 year up to 18 years. Present ambulatory status stretched from non-ambulatory to no restrictions at all. For additional clinical information as well as patients’ variants detected in their *DMD* genes, see Table 1, and for detailed case presentations, see Supplementary Document S1.

### 3.3. Muscle Biopsy Findings

**Figure 2:**
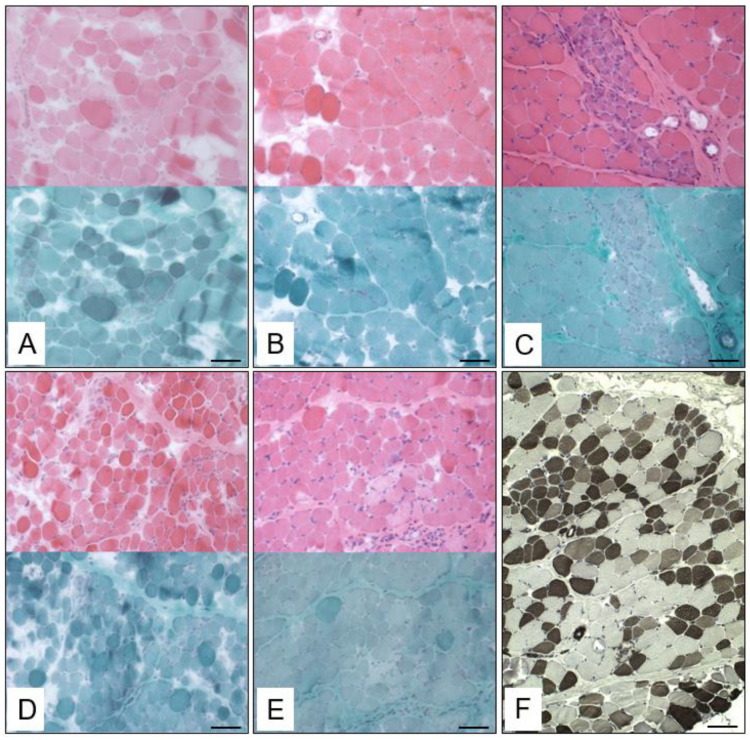
Histological studies showing dystrophic features in muscle biopsy specimens of patient 2 (**A**), patient 4 (**B**), patient 5 (**C**), patient 9 (**D**), patient 10 (**E**) and patient 8 (**F**). (**A**–**E**) Hematoxylin and eosin stains (upper panels) and Gomori trichrome stains (lower panels) and (**F**) ATPase stain at pH 4.3. Scale bars = 200 µm.

The available muscle biopsies (9/13 patients) were examined microscopically and showed the classical dystrophic tissue pattern characterized by inflammation and fibrosis in all cases (see Table 2). On the histological level, a predominance of type 1 fibers was visible in 2/10 cases, whereas a predominance of type 2 fibers was identified in 2/10 cases. Fiber regeneration was present in 5/10 cases and fascicular structure was visible in all biopsies. The proliferation of perimysial connective tissue was visible in 6/10 cases and adipocyte proliferation was visible in 4/10 cases. A variability in fiber size was in turn present in all biopsies, whereas group formation was seen in 5/10 cases. Phagocytosis was present in 4/10 cases, while cell necrosis was present in 7/10 cases. Intracellular glycogen or lipid proliferation was not visible in any of these biopsies. These microscopic findings are shown for paradigmatic cases in Figure 2: histological studies of patient 2 revealed a dystrophic pattern, with fiber regeneration, variability in fiber size, proliferation of connective tissue and adipocytes as well as cell necrosis being present, whereas based on the clinical presentation, one would have assumed a less pronounced appearance. The same applied to patient 4, with muscle biopsy showing a dystrophic pattern as well as the proliferation of perimysial connective tissue, but a rather mild phenotype. The histological findings of patient 5 indicated no signs of fiber regeneration, but muscle fiber necrosis, proliferation of connective tissue and adipocyte proliferation. Histological studies on the muscle biopsy derived from patient 9 in particular unveiled a highly significant variability in fiber size and myofiber necrosis.

**Figure 3:**
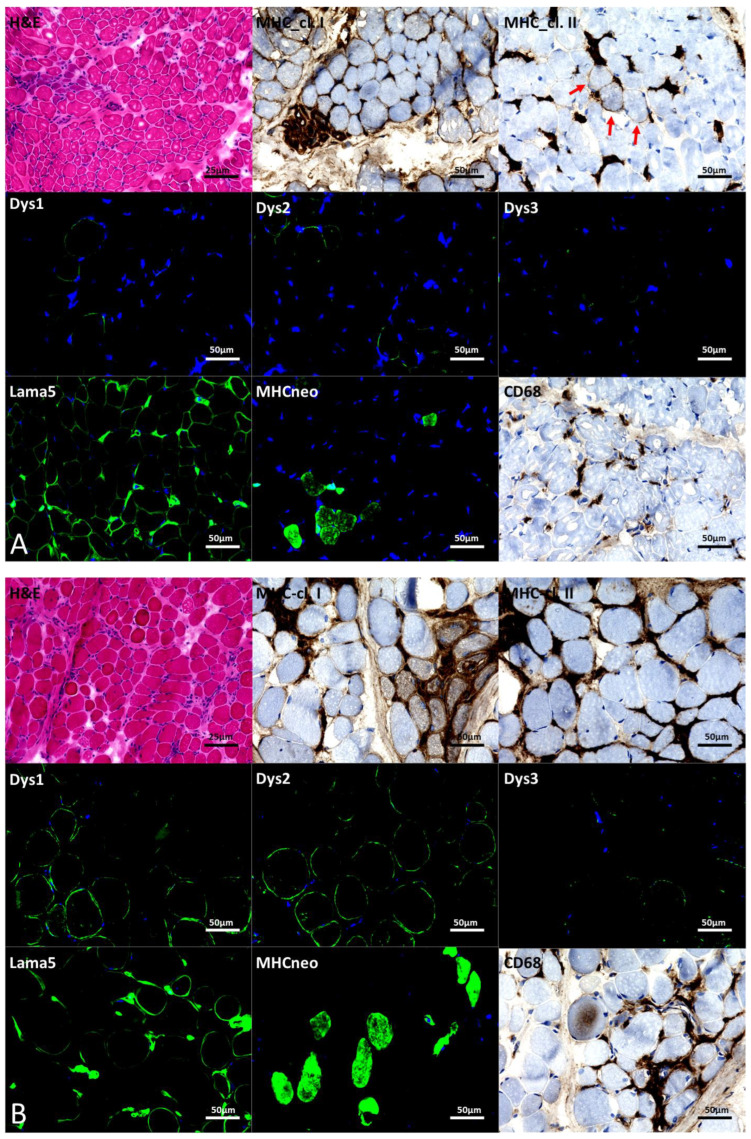
Microscopic studies of patient 1 (**A**) and patient 11 (**B**) as two dystrophinopathy cases of our cohort presenting with different clinical severities. (**A**) H&E showing a dystrophic pattern accompanied by endomysial infiltration and fibrosis. IHC staining revealed MHC class I immunoreactivity on multiple muscle fibers (regenerating fibers). MHC class II was negative on muscle fibers but found on immune cells (e.g., macrophages) and physiologically on capillaries. IF (Dys 1–3) showed a decrease in the expression of Dys 1–Dys 3. IF of LAMA5 showed a moderate increase in expression. MHCneo expression was strongly increased, indicative of active muscle fiber regeneration. IHC of CD68+ showed macrophages invading the muscle and building cluster with further immune cells. (**B**) H&E showed a dystrophic pattern accompanied by infiltrating immune cells and fibrosis. IHC showed that MHC class I was positive in revertant fibers (regenerating fibers, small, clustered), while MHC class II was found only in singular muscle fibers (red arrow) in addition to the physiological expression on capillaries. IF (Dys 1–3) showed loss of DMD expression except in some revertant fibers. IF of Lama5 showed sarcolemmal increase and an IF of MHCneo revealed an increase in regenerating fibers.

An increase in major histocompatibility complex (MHC) class I (while MHC class II was found only in singular muscle fibers) in addition to an increase in LAMA5 and CD68 was also present in all biopsies as shown for paradigmatic cases (patients 1 and 11) in Figure 3.

## 4. Discussion

Here, we performed a clinical, molecular genetic and microscopic study of a large monocentric cohort of dystrophinopathy patients harboring variants in their *DMD* gene and identified 13 putative disease-causing *DMD* small variants which had not been previously described in the literature, thus expanding the current genetic landscape of dystrophin-associated muscle diseases. Muscle biopsies were available for microscopic analyses in 10 patients and enabled insights into the associated myopathology.

Out of 13 patients, five were overall classified as DMD and three were classified as BMD. Among them, nine were affected by variants in the central rod domain while the distal cysteine-rich domain was involved in one patient and C-terminal domains were involved in two patients. In one patient, the proximal actin-binding domain was involved.

### 4.1. Phenotype–Genotype Correlations

Nonsense or truncating variants usually lead to the interruption of dystrophin synthesis, followed by a degradation of the dystrophin protein (or a decay of the mutant transcript already escaping translation as a general note, an event known as nonsense-mediated decay). Truncating variants in the central rod domain (CRD) are often predicted to result in a more severe DMD phenotype compared to those affected N-terminal and C-terminal exons, with a few exceptions reported [37,38,39]. Underlying mechanisms to these exceptions may be the initiation of translation downstream from ATG codons (serving for nonsense mutations near the 5′ gene region) or an inefficiently working nonsense-mediated decay (NMD) or some central in-frame exons, with truncating variants undergoing spontaneous exon skipping [37,38,39]. In our cohort, out of our clinically DMD patients, two (8 and 9) carried frameshift (c.7093delG; p.Val2365Leufs*6) and nonsense (c.7484C>G; p.Ser2495*) variants in the distal hotspot central rod domain (CRD) exons 48 and 51, respectively. Frameshift variants in CRD exons were also identified in three patients (3, 6 and 10) whose phenotype severity cannot be finally classified as of now although they are expected to be of the DMD phenotype. Patient 10 having a complete absence of dystrophin staining further supports this correlation.

Among our patients, three (4, 5 and 7) also had canonical splice site variants affecting the abnormal splicing of CRD exons 20, 29 and 47, respectively (Table 1). However, out of these, only patient 4 was clinically confirmed to have the DMD phenotype, while the phenotype severity in the other two is yet to be determined. Splice site variants have been historically reported more commonly in BMD patients although the predicted phenotype can vary depending on various factors like residual normal splicing, effect of mis-splicing events leading to either exon skipping or pseudoexon retention and whether the reading frame of RNA is maintained [40,41,42]. In patients 4 and 5, near complete loss of dystrophin staining in the muscle aligned with the DMD phenotype. However, further RNA analysis might be required to determine the exact impact of these splicing variants.

Patients 12 and 13 had predicted truncations affecting their distal C-terminal exons 74 and 78. In patient 12, the frameshift in exon 74 caused premature truncation after 21 codons (p.Leu3471Phefs*21), which effectively results in the loss of five distal exons. In patient 13, the deep intronic variant was shown to cause partial inclusion of intron 77 and premature truncation, effectively causing deletion of exons 78 and 79. Premature stop codons in the 3′ end *DMD* exons 72–76, which are alternatively spliced, have been associated with partial functional truncated protein and milder phenotypes [39]. Terminal truncating variants can avoid nonsense-mediated decay and complete loss of protein, as previously reported [38]. Hence, these findings correlate with the BMD phenotype observed in patients 12 and 13. It was also shown that distal *DMD* deletions were associated with neurodevelopmental symptoms along with a less severe muscle phenotype [38,43,44,45]. This is compatible with the phenotype of patient 13, including milder muscle findings and autism. Indeed, the results of our immunostaining studies showed a residual abundance of Dys 2, a microscopic finding which is in line with the predicted and in fact presenting phenotype of this patient.

Further, patient 2 was reported to have a deep intronic variant, which resulted in pseudoexon formation and premature truncation (p.Gly384Leufs*3) distal to exon 10. While such a proximal truncation was expected to most likely result in the total loss of dystrophin, the scores of the cryptic donor and acceptor splice sites predicted by SpliceAI were of lower strengths at 0.26 and 0.28, respectively, which indicated the possibility of leaky splicing associated with the partial disruption of a normal transcript and hence a milder phenotype [34]. This in turn indicated that these predictions needed to be considered with caution, an important aspect not only in the counselling of parents/caregivers but also in the decision making regarding appropriate therapeutic intervention concepts.

Pathogenic amino acid substitutions in terms of missense variants count for up to <1% of dystrophinopathies [17] and can lead to either DMD or BMD [46] based on their pathogenic impact of the DMD protein structure and stability. In two missense variants in our cohort (patients 1 and 11), we predicted a possible mis-splicing effect, which might have explained the pathogenicity of these variants, although further functional validation might be necessary. In patient 1, the in-frame INDEL c.336_337delinsTT was predicted to alter the normal splicing of exon 5, which was an in-frame exon, by creating a significant imbalance in SREs (ESE/ESS: -12). Further, truncations or nonsense mutations in proximal N-terminal exons including exon 5 have been associated with the BMD phenotype [39]. This may have explained the milder phenotype in patient 1, which also matched the only moderately pronounced dystrophic biopsy findings and partially reduced dystrophin staining. On the other hand, patient 11, who also had a rare missense (c.9527A>G) in distal exon 65, presented with a severe DMD phenotype. However, in silico predictions showed a high likelihood of a cryptic donor site activation due to this substitution, which could have resulted in a partial deletion of exon 65 and loss of reading frame. This further correlates with the near total loss of dystrophin staining in the muscle.

### 4.2. CNS Abnormalities and Correlation with Location of Variants

Distal *DMD* transcripts of Dp71 and Dp140 expressed in the brain have been associated with brain development [47]. Patients with pathogenic variants affecting the loss of these distal transcripts have been linked to more severe neuropsychiatric abnormalities [48,49]. In the current cohort, while patients 11 to 13 had variants affecting their distal Dp71, patients 7–10 were identified as having variants distal to the transcriptional start site of Dp140. Of note, in patients 11 and 13, global developmental delay as well as autistic behavior was reported, clinical findings which accord with the findings of Thangarajh et al. and Darmahkasih et al., stating that distally located variants are more likely to develop autistic behavior [43,44]. The learning disability of patient 1 may be explained by the proximal location of his two variants, localized in the actin-binding domain, since proximal variants are associated with learning disabilities according to the results of Banihani et al. [50].

One might consider that instead of making use of muscle biopsies, *DMD* quantification may have been carried out on white blood cells, especially for the four cases included in our study, for which no muscle biopsies were available. This hypothesis is supported by the results of mRNA studies in a cohort of DMD patients which unveiled the opportunity of direct detection of *DMD* rearrangements by the investigation of peripheral blood lymphocytes [51]. However, these biopsies were mostly historical and not all variants may have affected the DMD isoform expressed within these cells, serving as an alternative in vitro model. A latter aspect is supported by the findings of another study which demonstrated that the analysis of *DMD* mRNA expression from skeletal muscle but not from lymphocytes led to the identification of a novel nonsense mutation in a carrier of dystrophinopathy [52]. Hence, more sophisticated analytical approaches such as targeted proteomics would be crucial to deciphering the suitability of white blood cells to correlate the DMD protein (isoform) level with different *DMD* defects, especially in light of their localization [53].

Moreover, reference studies on a larger cohort of dystrophinopathy patients with different genotypes and associated phenotypes would have been crucial to be included in this study to draw final conclusions, but this biomaterial was not available. However, apart from the lack of muscle biopsies derived from all patients highlighted in this study, our retrospective study also harbored further different limitations: one possible limitation in the evaluation of the findings could have been a different assessment of the remaining walking distance, as this information was given subjectively. In addition, the information was not directly comparable, as some patients gave walking distances, while others gave the remaining possible duration. In addition, the different courses of the disease were not to be regarded as typical for the variants, as all cases were new descriptions without comparable cases.

## 5. Conclusions

In summary, our clinical as well as our genetic findings support the concept of a clinical continuum of dystrophinopathies rather than the restrictive division into DMD and BMD. Moreover, our combined data highlight that it remains challenging to deduce the course of disease from genetic analyses alone. Though the reading frame rule may be applicable to most pathogenic variants, there still are exceptions to the rule and some patients who cannot be reliably assigned to either a DMD or a BMD but show courses of disease in between, fitting in the concept of dystrophinopathies as a more appropriate nomenclature. For example, rather early symptoms are associated with a long-preserved walking ability. In addition, the effects of any diagnosis on the family should be considered, whereby an incorrect assumption regarding the clinical course of disease may have extensive consequences, no matter whether regarding a false diagnosis of DMD or BMD.

Overall, our findings support the assumption that only clinical features, molecular findings and DMD quantification on biomaterial (such as muscle biopsy) taken together may serve as prognostic predictors of the severity of disease in between the continuum of DMD and BMD. Hereby, a muscle biopsy harbors the benefit of also enabling drawing conclusions upon a widespread myopathology in terms of myodegeneration.

## Figures and Tables

**Table 1:** Overview of clinical and molecular genetic findings of 13 patients with novel DMD variants. h = hours; IQ = Intelligence quotient; km = kilometers; LOA = loss of ambulation; m = meters; min = minutes; NA = not applicable; ND = not done; Unk = unknown.

Patient Number	Present Age (in Years)	Present Ambulatory Status	Age of Onset (in Years)	Type of First Symptom	Exon/Intron	Pathogenic Variant in the *DMD* Gene	Protein Change	Interpretation	Prediction Based on Genetic Findings	Age at Muscle Biopsy (in Years)	CK at Muscle Biopsy (Ref.Range < 165 U/L)	CNS Involvement	Cardiac Involvement	Ventilatory Support
1	18	0.5 h Walking possible	2	Motor delay	Exon 5	c.336G>T, c.337A>T	p.Trp112Cys, p.Asn113Tyr	Missense, Missense	BMD	4	11,474	Learning disability (IQ 70)	Mild left ventricular dilatation	Not needed
2	17	100 m Walking possible	0.5	Frequent falls	Intron 10	c.1149+273T>G	p.Gly384Leufs*3	Nonsense	DMD	3	38,493	None	None	Not needed
3	12	1 h Walking possible	Unk	Unk	Exon 17	c.2041_2042delGT	p.Val681Asnfs*38	Nonsense	DMD	ND	NA	None	Left ventricular ejection fraction 50%	Not needed
4	16	100–150 m Walking possible	4	Motor delay	Intron 19	c.2381-2A>T	p.?	Splice site	DMD	5	10,464	Speech delay, motor tics	None	Not needed
5	10	0.5–1 h Walking possible	4	Motor delay	Exon/Intron 29	c.4071+1delG	p.?	Splice site	UNK	4	7187	Speech delay	None	Not needed
6	9	3–4 km Walking possible	4	Motor delay	Exon 39	c.5516_5517del	p.Thr1839Argfs*2	Nonsense	DMD	ND	NA	Difficulties concentrating	Mild left ventricular dilatation	Not needed
7	7	No limitations	5	Motor delay	Intron 47	c.6912+2T>C	p.?	Splice site	BMD	ND	NA	None	None	Not needed
8	14	LOA 7	3	Motor delay	Exon 48	c.7093delG	p.Val2365Phefs*6	Nonsense	DMD	4	26,433	None	Left ventricular dilatation	Not needed
9	12	Ambulatory at home	3	Motor delay	Exon 51	c.7484C>G	p.Ser2495Stop	Nonsense	DMD	1	18,332	Mild intellectual disability (IQ 60)	None	Not needed
10	1	No limitations	1	Motor delay	Exon 59	c.8890_8891dup	p.Asp2965Alafs*25	Nonsense	UNK	1	18,308	None	None	Not needed
11	7	10–15 min Walking possible	1	Motor delay	Exon 65	c.9527A>G	p.Asp3176Gly	Missense	DMD	2	11,731	Autistic behavior, global developmental delay	None	Not needed
12	18	No limitations	10	Motor delay	Exon 74	c.10406_10409dup	p.Leu3471Phefs*21	Nonsense	BMD	ND	NA	None	None	Not needed
13	10	3–4 km Walking possible	1	Motor delay	Intron 77	c.11015-545A>G	p.?	Splice site	BMD	3	2800	Autistic behavior, global developmental delay	None	Not needed

**Table 2:** Myopathological findings in biopsy specimens. Dys = dystrophin; MQFR = musculus quadriceps femoris (right); MVLR = musculus vastus lateralis (right); Neg. = negative; ND = not done; Unk = unknown; − = not present + = present ++ = very present − − = strongly reduced +/– = mildly reduced.

Patient Number	Muscle	Fiber Type Predominance	Lymphocyte Infiltrates	Fiber Regeneration	Fascicular Structure Preserved	Perimysial Connective Tissue	Adipocyte Proliferation	Variability in Fiber Size	Group Information	Centralized Cell Nuclei	Phagocytosis	Cell Necrosis	Intracellular Glycogen or Lipid Proliferation	Dys 1	Dys 2	Dys 3
1	MQFR	Both	++	+	+	+	+	+	+	<3%	+	+	−	+/−	+/−	+/−
2	MQFR	Both	Unk	+	+	+	+	++	Unk	<3%	Unk	+	−	− −	− −	− −
3	ND	ND	ND	ND	ND	ND	ND	ND	ND	ND	ND	ND	ND	ND	ND	ND
4	MVLR	Both	+	−	+	+	−	+	Unk	<3%	+	−	−	− −	− −	Neg.
5	MVLR	Type 1 fibers	Unk	Unk	+	+	−	+	+	<3%	+	+	−	− −	− −	Neg.
6	ND	ND	ND	ND	ND	ND	ND	ND	ND	ND	ND	ND	ND	ND	ND	ND
7	ND	ND	ND	ND	ND	ND	ND	ND	ND	ND	ND	ND	ND	ND	ND	ND
8	MVLR	Type 2 fibers	+	−	+	+	+	++	Unk	<3%	Unk	+	−	Neg.	Neg.	Neg.
9	MQFR	Type 1 fibers	+	+	+	−	−	++	+	<3%	Unk	++	−	Neg.	Neg.	Neg.
10	MVLR	Both	−	+	+	−	+	+	+	<3%	+	+	−	Neg.	Neg.	Neg.
11	MVLR	Both	+	++	+	−	−	+	+	<3%	Unk	++	−	− −	− −	Neg.
12	ND	ND	ND	ND	ND	ND	ND	ND	ND	ND	ND	ND	ND	ND	ND	ND
13	MVLR	Type 2 fibers	−	−	+	+	+	Physio−logical	−	<3%	−	−	−	+	Neg.	− −

## Data Availability

The original contributions presented in this study are included in the article and Supplementary Materials. Further inquiries can be directed to the corresponding author, Andrea Gangfuß.

## Supplementary Materials

The following supporting information can be downloaded at: https://www.mdpi.com/article/10.3390/biomedicines12122738/s1, Supplementary Figure S1: Inserted sequence (pseudoexon) including exact boundaries between exons 10 and 11 of patient 2. Supplementary Figure S2: RNA-Seq analysis of patient 13. Supplementary Document S1: Detailed case reports. Supplementary Document S2: Methodology of RNA sequencing in patients 2 and 13. Supplementary Table S1: Patient classification.
