# Supplementary material for: Expanding the Molecular Genetic Landscape of Dystrophinopathies and Associated Phenotypes
Source: Biomedicines. 2024 Nov 29;12(12):2738. doi: 10.3390/biomedicines12122738 (PMC11727156; doi:10.3390/biomedicines12122738)
Supplement: Supplementary file 1 [file biomedicines-12-02738-s001.zip › Supplementary Document S1.pdf]

Sequencing of the DMD gene of **patient 1** showed a 2bp inframe INDEL (c.336_337delinsTT) in exon 5 predicting a BMD phenotype. He does not take medication influencing the course of disease, still being able to walk 30 minutes at the age of 18 years, indicating that the prediction of BMD based on genetic findings might be correct.

Patient 2 who was identified to have a novel intronic variant c.1149+273T>G affecting a premature truncation (p.Gly384Leufs*3) distal to exon 10 in proximal rod domain predicting DMD, was able to walk without support at the age of 21 months. Currently, he is 17 years old and able to walk 100 metres and is under treatment with Deflazacort since the age of 7. Of note, ventilatory support is not needed at present and cardiac involvement has not been observed yet. No signs of CNS involvement to benoted.

Sequencing of the DMD gene in **patient 3** was carried out at the age of 4 years and revealed the novel variant c.2041_2042delGT predicting DMD. Currently he is 12 years old and able to walk one hour and is under treatment with Deflazacort since the age of 6. Last cardiac exam showed a reduced left ventricular ejection fraction of 50%. The boy is currently too young to be definitively categorised as BMD or DMD.

Sequencing of the DMD gene of **patient 4** at the age of 5 years showed the novel variant c.2381-2A>T predicting DMD. He is taking Deflazacort since the age of 6, being able to walk up to 150 meters at the age of 16 years, indicative for a DMD phenotype from the milder end of the spectrum. Cardiac examination shows structural normal findings, CNS involvement shows in speech delay as well as motor tics. He does not need ventilatory support.

In **patient 5** sequencing of the DMD gene was initiated at the age of 4 years and revealed the novel variant c.4071+1delG with uncertain effects on the phenotype. First symptoms was delayed development of fine motor skills at the age of 4. He achieved free walking at the age of 10 months. At the age of 10 years, he has a maximal walking distance of up to one hour. Last cardiac exam was normal. He does take Deflazacort since the age

of 7. The boy is currently too young to be finally categorised as BMD or DMD.

Sequencing of the DMD gene in **patient 6** was performed at the age of 4 years and revealed the novel variant c.5516_5517del predicting DMD. He was able to walk unaided at the age of 14 months and showed a motor delay at the age of 4 years. At an age of 9 years, he is able to walk up to 4 km unaided. He receives Deflazacort since the age of 6. Cardiac exam showed a mild left ventricular dilatation at 8 years of age. He reports of difficulties in concentrating in school. The boy is currently too young to be definitively categorised as BMD or DMD.

Sequencing of the DMD gene of **patient 7** at the age of 6 years showed the novel variant c. c.6912+2T>C predicting BMD . First symptom was muscle pain at the age of 5. He did not take any medication influencing the course of disease, being able to walk without limitations at the age of currently 7 years. The boy is currently too young to be definitively categorised as BMD or DMD.

Sequencing of the DMD gene in **patient 8** was carried at the age of 4 years and revealed the novel variant c.7093delG, predicting DMD. He was able to walk unaided at the age of 19 months and showed a motor delay at the age of 3 years. Loss of ambulation occurred at 7 years of age, taking into account that he received Deflazacort for only one year (6-7 years of age). Cardiac exam shows a left ventricular dilatation at currently 14 years of age. There are no symptoms indicating CNS involvement. In summary, a severely affected DMD phenotype.

Sequencing of the DMD gene in **patient 9** was carried at the age of 4 years and revealed the novel variant c.7484C>G, predicting DMD. He was able to walk unaided at the age of 16 months and showed exercise intolerance at the age of 3 years. At an age of 12 years, he is able to walk only a few steps unaided. He receives Deflazacort since the age of 5, as well as Translarna. His last cardiac exam showed normal results. He does not need ventilatory support. Taken together, clinical findings point toward a DMD phenotype.

In **patient 10** sequencing of the DMD gene was initiated at the age of 6 months and revealed the novel variant c. 8890_8891dup. First symptoms was an abnormal gait before reaching free walking ability at the age of 16 months. Treatment has not yet been initiated and the course of the disease is still uncertain.

Sequencing of the DMD gene of **patient 11** at the age of 2 years showed the novel variant c. c.9527A>G predicting DMD. First symptom was a motor delay at the age of 1. He does take Deflazacort since the age of 5, being able to walk 10-15 minutes at the age of currently 7 years, pointing towards a DMD phenotype. CNS involvement includes a global developmental delay and autistic behaviour.

In **patient 12** sequencing of the DMD gene was initiated at the age of 14 years and revealed the novel variant c.10406_10409dup in C-terminal exon 74 predicting BMD. First symptoms were exercise-induced muscle pain at the age of 10. He reached free walking ability at the age of 15 months. At the age of 18 years, he has an age-appropriate maximal walking distance, goes to the gym regularly and reports muscle pain only after drinking alcohol. There are no symptoms indicating CNS involvement. These clinical observations show a clinical course consistent with BMD.

Sequencing of the DMD gene of **patient 13** at 7 years of age displayed the novel intronic variant c.11015-545A>G, RNASeq analysis revealed insertion of a pseudo exon, predicting BMD. First presentation at our clinic was at the age of 11 months due to dystrophy (weight 6590g, <1st percentile) and short stature (length 69 cm, 1st percentile) as well as elevated creatine kinase levels and motor developmental delay. At 10 years of age, he is able towalk 3 kilometres. He was never able to run or jump but can climb stairs when using the handrail. A global developmental delay with autistic behaviour was diagnosed at six years of age, due to which a standardised IQ-testing was never possible to be performed. In summary, the clinical course is consistent with a BMD phenotype.
