# Supplementary material for: Expanding the Molecular Genetic Landscape of Dystrophinopathies and Associated Phenotypes
Source: Biomedicines. 2024 Nov 29;12(12):2738. doi: 10.3390/biomedicines12122738 (PMC11727156; doi:10.3390/biomedicines12122738)
Supplement: Supplementary file 1 [file biomedicines-12-02738-s001.zip › Supplementary Document S2.pdf]

For cDNA analysis of patient 2 RNA form muscle tissue was isolated using RNeasy Mini (Qiagen, Hilden, Germany) and cDNA was synthesized with SuperScript II RT (Thermo Fisher Scientific, Waltham, Massachusetts, USA). Overlapping cDNA fragments of the entire DMD gene were amplified and the fragment comprising exons 6-12, which showed an augmented fragment size, was sequenced using BigDye Terminator sequencing mix v1.1 on an ABI3130xl Genetic Analyzer (Thermo Fisher Scientific, Waltham, Massachusetts, USA).

In patient 13, RNA was extracted from muscle tissue and the TruSeq Stranded mRNA Library Prep Kit (Illumina) was used to create RNA-Seq libraries. Libraries were sequenced on a NovaSeq 6000 (Illumina). As a reference genome, the GRCh37 primary assembly (GENCODE release 34) was used. Detection of aberrant splicing was based on DROP v1.3.3.
