# Supplementary material for: Expanding the Molecular Genetic Landscape of Dystrophinopathies and Associated Phenotypes
Source: Biomedicines. 2024 Nov 29;12(12):2738. doi: 10.3390/biomedicines12122738 (PMC11727156; doi:10.3390/biomedicines12122738)
Supplement: Supplementary file 1 [file biomedicines-12-02738-s001.zip › Supplementary Figure S1.pdf]

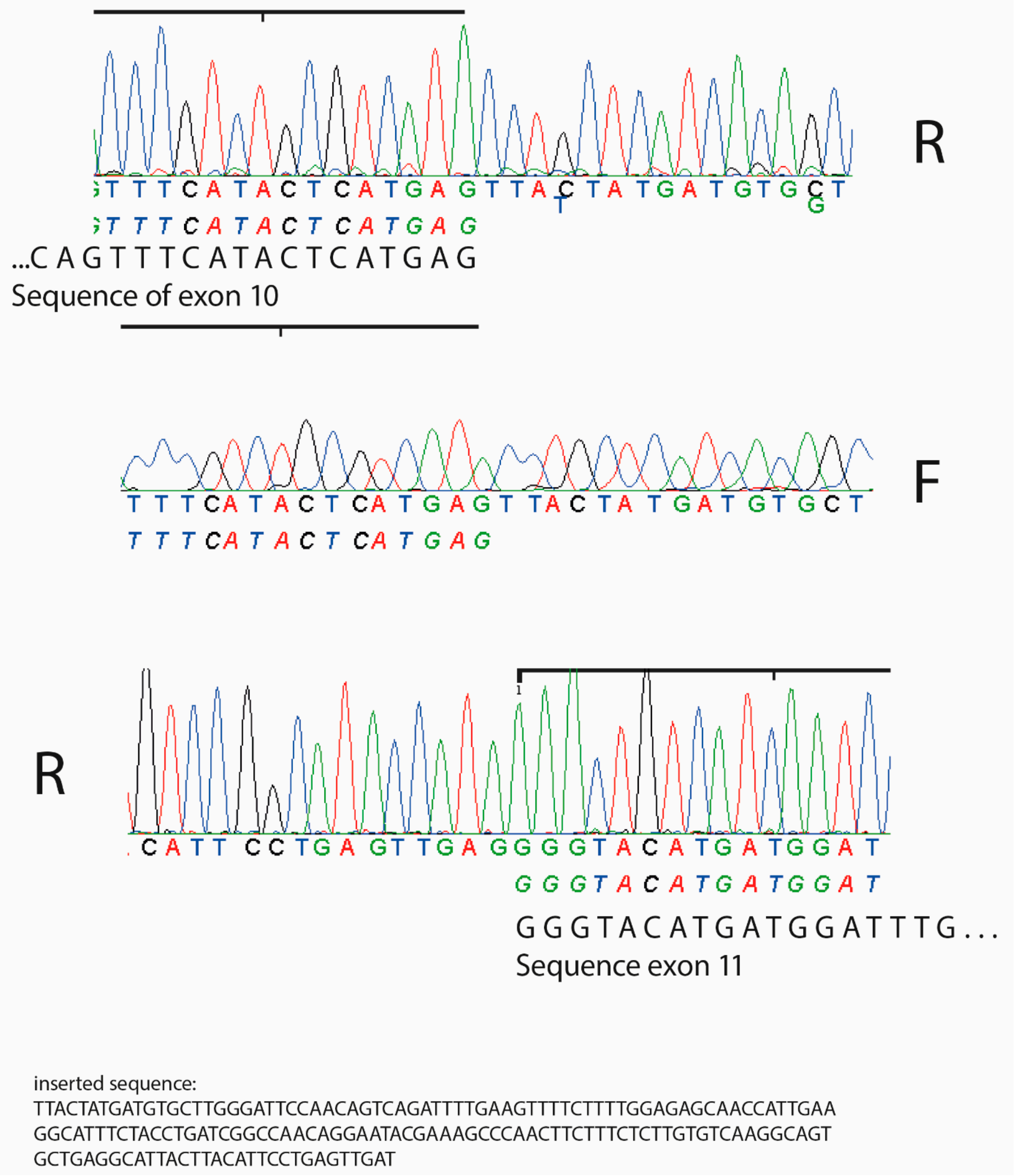

Supplementary Figure S1 Inserted sequence (pseudoexon) including exact boundaries between exon 10 and 11 of patient 2.
