# Supplementary material for: Expanding the Molecular Genetic Landscape of Dystrophinopathies and Associated Phenotypes
Source: Biomedicines. 2024 Nov 29;12(12):2738. doi: 10.3390/biomedicines12122738 (PMC11727156; doi:10.3390/biomedicines12122738)
Supplement: Supplementary file 1 [file biomedicines-12-02738-s001.zip › Supplementary Figure S2.pdf]

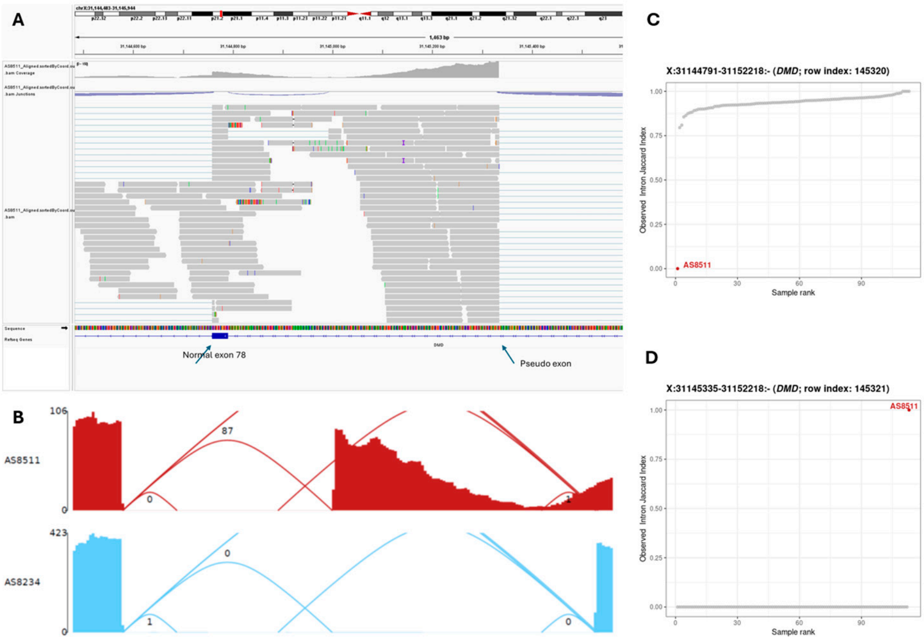

Supplementary Figure S2 RNAseq analysis of patient 13. (**A): **Integrative genomics viewer (IGV) indicating the normal exon and the pseudo exon. (**B): **Sashimi plot showing abnormal splicing with pseudo exon inclusion due creation of a cryptic acceptor site in intron 77. Jaccard index plots showing reduced splicing of the canonical splice junctions of intron 77 (**C**) while showing increased expression of newly created splice junction (**D**).
